# Systemic Administration of Fibroblast Growth Factor 21 Improves the Recovery of Spinal Cord Injury (SCI) in Rats and Attenuates SCI-Induced Autophagy

**DOI:** 10.3389/fphar.2020.628369

**Published:** 2021-01-27

**Authors:** Sipin Zhu, Yibo Ying, Lin Ye, Weiyang Ying, Jiahui Ye, Qiuji Wu, Min Chen, Hui Zhu, Xiaoyang Li, Haicheng Dou, Huazi Xu, Zhouguang Wang, Jiake Xu

**Affiliations:** ^1^ Department of Orthopaedics, The Second Affiliated Hospital and Yuying Children’s Hospital of Wenzhou Medical University, Wenzhou, China; ^2^ School of Biomedical Sciences, The University of Western Australia, Perth, WA, Australia; ^3^ The Second School of Medicine, Wenzhou Medical University, Wenzhou, China; ^4^ Spinal Cord Injury Treatment Center, Kunming Tongren Hospital, Kunming, China; ^5^ Department of Molecular Pharmacology, Albert Einstein College of Medicine, Bronx, NY, United States

**Keywords:** spinal cord injury, fibroblast growth factor 21, autophagy, nerve regeneration, fibrotic scar

## Abstract

Protecting the death of nerve cells is an essential tactic for spinal cord injury (SCI) repair. Recent studies show that nerve growth factors can reduce the death of nerve cells and promote the healing of nerve injury. To investigate the conducive effect of fibroblast growth factor 21 (FGF21) on SCI repair. FGF21 proteins were systemically delivered into rat model of SCI via tail vein injection. We found that administration of FGF21 significantly promoted the functional recovery of SCI as assessed by BBB scale and inclined plane test, and attenuated cell death in the injured area by histopathological examination with Nissl staining. This was accompanied with increased expression of NeuN, GAP43 and NF200, and deceased expression of GFAP. Interestingly, FGF21 was found to attenuate the elevated expression level of the autophagy marker LC3-II (microtubules associated protein 1 light chain 3-II) induced by SCI in a dose-dependent manner. These data show that FGF21 promotes the functional recovery of SCI via restraining injury-induced cell autophagy, suggesting that systemic administration of FGF21 could have a therapeutic potential for SCI repair.

## Introduction

Spinal cord injury (SCI) is a devastating neurological disorder resulting in the loss of motor and sensory function (Taccola et al., 2018; Li et al., 2019). Due to the non-reproducibility of neurons, the natural self-repair from SCI is very limited (Zholudeva et al., 2018). SCI can be operationally divided into primary injury initiated by mechanical impact, and secondary injury caused by autophagy, inflammation, apoptosis, oxidative stress and other factors (Kim et al., 2018; Ko et al., 2020; Vismara et al., 2020). In SCI, following the primary injury, a large number of nerve cells are damaged by subsequent secondary injury, resulting in severe functional loss (Qian et al., 2020; Zhang et al., 2020). The damaged nerve fiber degeneration, local edema, ischemia and hypoxia caused by SCI will lead to a series of secondary injury reactions such as anaerobic metabolism, tissue acidosis and free radical reaction, which could promote the apoptosis and autophagy of spinal cord neurons due to the hypoxia microenvironment, resulting in continuous impairment of spinal cord function (Lipinski et al., 2015; Gonzalez Porras et al., 2018; Abbaszadeh et al., 2020). Therefore, the suppression of secondary SCI to reduce the death of neurons has become a key step for SCI repair therapy.

Autophagy is an important process of secondary SCI and has become a hotspot in SCI repair research (Li et al., 2019; Wu and Lipinski, 2019). Under physiological condition, autophagy is characterized by “cellular self-digestion” of autophagosomes with bilayer membrane structures in the cytoplasm (Schutter and Graef, 2020), and is beneficial to maintaining cell activity. However, in the pathogenesis of SCI, lysosomal injury and dysfunction could cause defects in autophagy flux, accumulation of autophagosomes and initiation of long-term and excessive-scale autophagic activity, which results in the damage of nerve cells (Rong et al., 2019). Microtubules associated protein 1 light chain 3-II (LC3-II), located on autophagy vesicles of mammalian cells is a specific marker of autophagy, and has been used to indicate the level of autophagy in cell (Malla et al., 2019). In the early stages of SCI, oxidative stress and apoptotic pathways are activated rapidly, mediating the death of spinal cord cells. In the chronic recovery period, autophagy is dominant. Although autophagy can clear damaged organelles in cells, overactivation of autophagy can also induce cell death. Therefore, inhibition of overactivative autophagy has become a key strategy for SCI recovery (Tang et al., 2015; Rong et al., 2019; Gu et al., 2020; Ko et al., 2020).

FGF21 is a member of FGF superfamily, which plays a role in regulating lipid and glucose balance and preventing metabolic disorders (Li, 2019; Keipert et al., 2020). In addition, FGF21 has been shown to promote nerve cell repair and regulate nerve development, survival and plasticity (Owen et al., 2014; von Holstein-Rathlou et al., 2016; Xiao et al., 2019). Interestingly, pancreas-derived FGF21 was found to promote the proliferation of oligodendrocyte precursor cells (OPC) and drive remyelination in the central nervous system (Kuroda et al., 2017). It was identified that OPCs expressed FGF21 coreceptor *β*-klotho, and knockdown of *β*-klotho expression in OPCs prevented the increase in OPC proliferation and remyelination (Kuroda et al., 2017). More recently, it has been reported that FGF21 can inhibit autophagy and promote the recovery of peripheral nerves (Lu et al., 2019), but the therapeutic effect of systemic administration of FGF21 on SCI and the role of FGF21 in SCI-induced autophagy remain unclear. In this study, FGF21 was administrated to SCI rats via tail vein injection to observe its therapeutic effect on the recovery of the damaged spinal cord. Here, we demonstrated for the first time that FGF21 promotes functional recovery from SCI, protects neurons and axons, and inhibits SCI-induced autophagy, and thus might serve as a promising molecule for the therapy of SCI repair.

## Materials and Methods

### Materials

Primary antibodies including neurofilament 200 (NF200, ab4680), glial fibrillary acidic protein (GFAP, ab7260), NeuN (ab104224), growth associated protein 43 (GAP43, ab75810), and LC3-II (ab192890), and secondary antibodies including goat anti-mouse 488 (ab150113), goat anti-rabbit 488 (ab150077), goat anti-chicken 488 (ab150169) and goat anti-rabbit tritc (ab6718) were purchased from Abcam (MC, United Kingdom). The DAPI was also under the supply of Abcam (MC, United Kingdom). Recombinant human FGF21 (rhFGF21) was obtained from Prof. Xiaokun Li, Zhejiang Provincial Key Laboratory of Biopharmaceuticals, Wenzhou Medical College, Wenzhou, Zhejiang, China. It was previously reported that rhFGF21 was produced using *Escherichia coli* and purified to be endotoxin free (Wang et al., 2010), and its biological effect further tested in nerve cells (Lu et al., 2019).

### Animal Model of SCI

Forty adult female SD rats were provided by the Animal Center of Chinese Academy of Sciences (Shanghai, China). The average weight of the rats is from 220 to 250 g at the time of surgery. Animal experiments were ethically approved by the Animal Care and Use Committee of Wenzhou Medical College (wydw2014-0074). The experiment was conducted according to the national institutes of health's guidelines for the care and use of laboratory animals. For experimental purposes, these rats were divided into four groups at random, including Sham group, SCI group, treatment group with 100 μg/ml FGF21, and treatment group with 500 μg/ml FGF21 (Lu et al., 2019). After 10% chloral hydrate (3.5 ml/kg, i. p.) anesthesia, SD rats were made an incision along the middle of the back skin to expose the eighth to 10th thoracic spinal vertebrae. By striking T9 segment of the spinal cord using a 10 g hammer and a 25-mm-height free fall, the acute injury of SCI model was generated. Animals in sham group underwent the same operation procedures except the collision damage. Animal care and treatment included bladder massage twice a day, in the morning and evening, to help SD rats expel urine.

### FGF21 Injection Through the Tail Vein to Treat SCI

Half an hour after the SCI model was established, 200 μl of 100 μg/ml FGF21 and 500 μg/ml FGF21 were injected into rats in different treatment groups through the tail vein, and then repeated every other 2 days. The Sham group was injected with saline alone at the same time. Tail vein injection was chosen to more readily deliver the drug into the site of SCI via blood circulation than intramuscular injection in this study. All the animals stayed at cage to recover. Same moderate diet was given to each group at fixed times.

### Locomotion Recovery Assessment

The Basso, Beattie and Bresnahan (BBB) locomotor rating scale scoring was conducted based on the natural process of exercise recovery of SCI rats, which ranges from 0 (responding to paralysis of the lower limbs) to 21 (reacting to normal motor function). The slanted test refers to a test device that was used to test and record the max angle at which rats could not fall and at least keep their position for 5 s. After wetting the rats’ hind feet with red dye, they were allowed to crawl through the suitable size box, then performing footprint scanning and digital image analysis.

### H & E Staining and Nissl Staining

To prepare samples for H & E staining, 10% chloral hydrate (3.5 ml/kg, i.p.) was applied to anesthetize the SD rats, and then thoracotomy was performed on the 60th day after injection. Rats were perfused with 0.9% NaCl, subsequently 500 ml paraformaldehyde phosphate buffer was injected into the heart to harden the tissue of rats. The spinal cord was removed at the eighth to 10th thoracic spinal vertebral level around the injury. The spinal cord was fixed overnight in 4% paraformaldehyde, and paraffin embedding was then performed. Hematoxylin and eosin (H & E) and Nissl staining was then performed to paraffin sections (5 μm thick) for histopathological examination.

### Video Imaging of Locomotor Function

Six female SD rats were randomly selected from each group for video imaging of locomotor function, including sham group, SCI group, 100 μg/ml FGF21 group and 500 μg/ml FGF21 group. Using a camera (Leica), rats of each group walked on a 1-m-long glass runway with marker on the hind limbs to take photos of the position of hips, knees, ankles and feet. Parameters to evaluate locomotion were listed as follows. Firstly, weight support, including height, hip height minus trunk width, equal to the torso gap on the ground. Secondly, leg extensor spasms (Quantifying the extent of extensor spasm by the time the foot is overstretched and dragged and by the relative duration of the legs on the back). Thirdly, the number of footsteps was recorded (Previous steps counted as reference). Fourthly, the posture of the foot (Measuring foot offset behind the hip at the beginning of the ankle) was also recorded. The front legs were used to determine the pace of walking steps (front leg steps/second) (Li et al., 2017).

### Immunofluorescence Staining

After pretreatment with xylene, and sodium citrate etc., the sections were incubated in PBS with 10% normal bovine serum and 0.1% Triton X-100 at 37°C for 1 h, and then with an appropriate primary antibody at 4°C overnight. The following primary antibodies against GFAP43 (1:500), NeuN (1:1000), NF200 (1:1000), GFAP (1:1000), LC3-II (1:500) were used. Next, the sections were washed with PBS for three times at room temperature. After that, the corresponding secondary antibodies (1: 500) were applied and incubated at 37°C avoiding light for 1 h. Sections were then washed 3 times with PBS, 5 min each time. Then DAPI (0.25 mg/ml) dye was applied for 7 min to allow nuclei staining. A Nikon ECLIPSE Ti microscope (Nikon, Tokyo, Japan) was used to take images. Fluorescent images were taken at the boundary between the normal area and the damaged area in comparable matching anatomical regions. Imagine J software was used to count the region of interest of at least three images and then SPSS13 software to process the obtained data to produce the statistical graph.

### Statistical Analysis

Statistical package for the social sciences (SPSS) analysis was used to evaluate the data which were expressed as mean ± SEM. When the experimental group was conducted in two groups, the student t test was applied to confirm the statistical significance. One-way analysis of variance (ANOVA) and Dunnett's post-mortem test were employed to evaluate the data when the comparison was conducted more than two groups. *p* < 0.05 was considered statistically significant.

## Results

### Systemic Administration of FGF21 Improves the Recovery of Spinal Cord Appearance and Hindlimb Function

**FIGURE 1:**
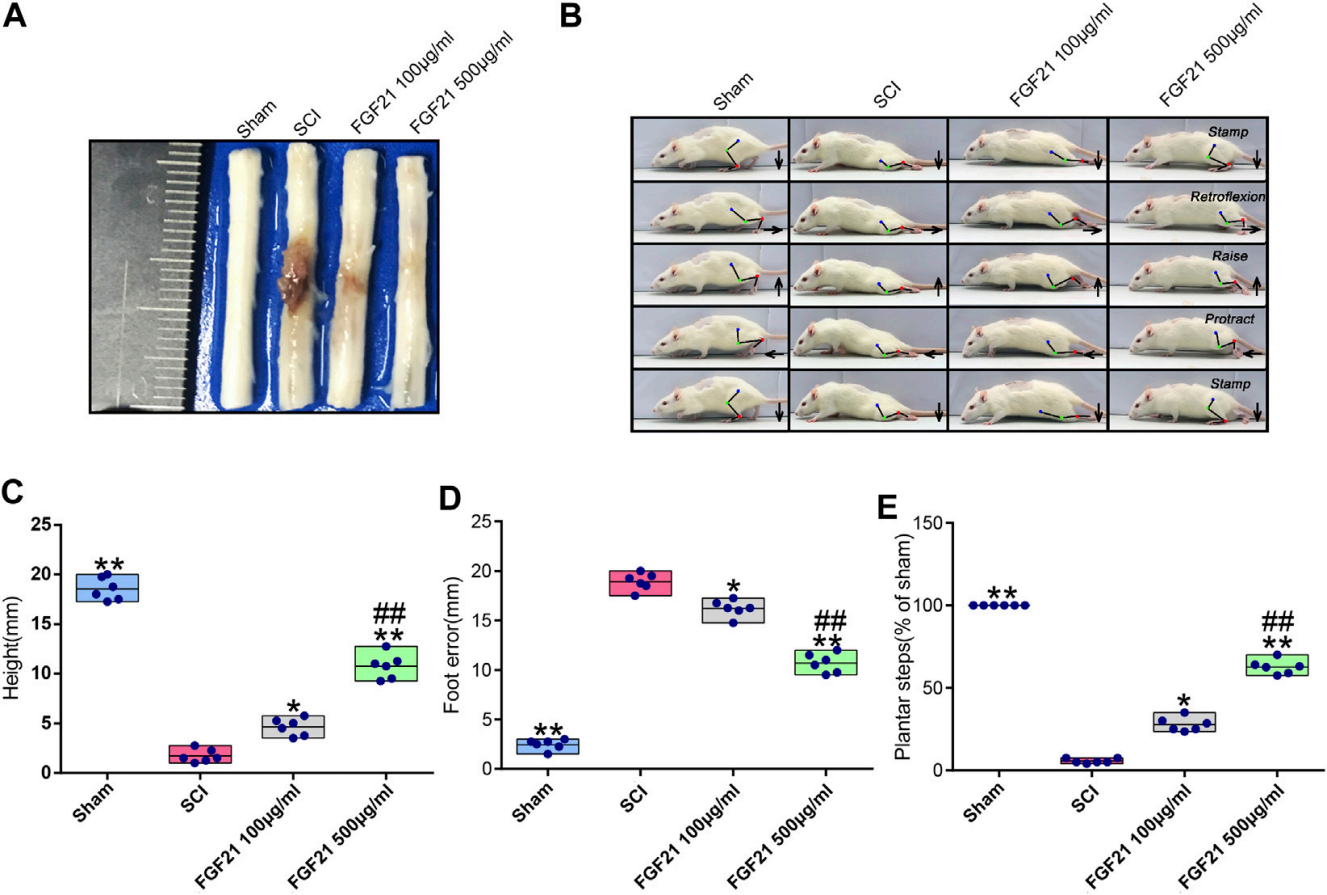
FGF21 treatment improves the recovery of spinal cord appearance and hindlimb function **(A)** Image to show different appearance of spinal cords in every group **(B)** Motional sequence of rats in every group. The blue, green and red dot marks represent Hip (iliac crest), knee and ankle joints; respectively. And they are connected by lines. Arrow displays the directions in which feet movement **(C–E)** Statistical graphs of height of trunk above the ground, foot-placement error and number of successful plantar steps. Representation of data is mean values ±SEM, *n* = 6, “*” and “**” represent *p* < 0.05 or *p* < 0.01 vs the SCI group, “##” represent *p* < 0.01 vs the 100 μg/ml FGF21 group.

To evaluate the treatment effect of FGF21 systemic injection on SCI, the visual images of morphology of spinal cords were taken. The spinal cord in the SCI group showed blackened and shrinked appearance, whereas in the group with systemic administration of FGF21, the spinal cord showed decreased color change and less atrophy (Figure 1A). In addition, video recording sequences revealed that the hind legs of SCI rats were stiff with no evident joint movement, whereas FGF21 treated groups showed varying degrees of improvement in hind leg movement as measured by height and plantar steps (Figure 1B). Further, when compared with SCI group, the footstep errors of the FGF21 treated groups were decreased as measured by the heights of trunk above the ground, foot-placement errors and number of successful plantar steps (Figures 1C–E).

**FIGURE 2:**
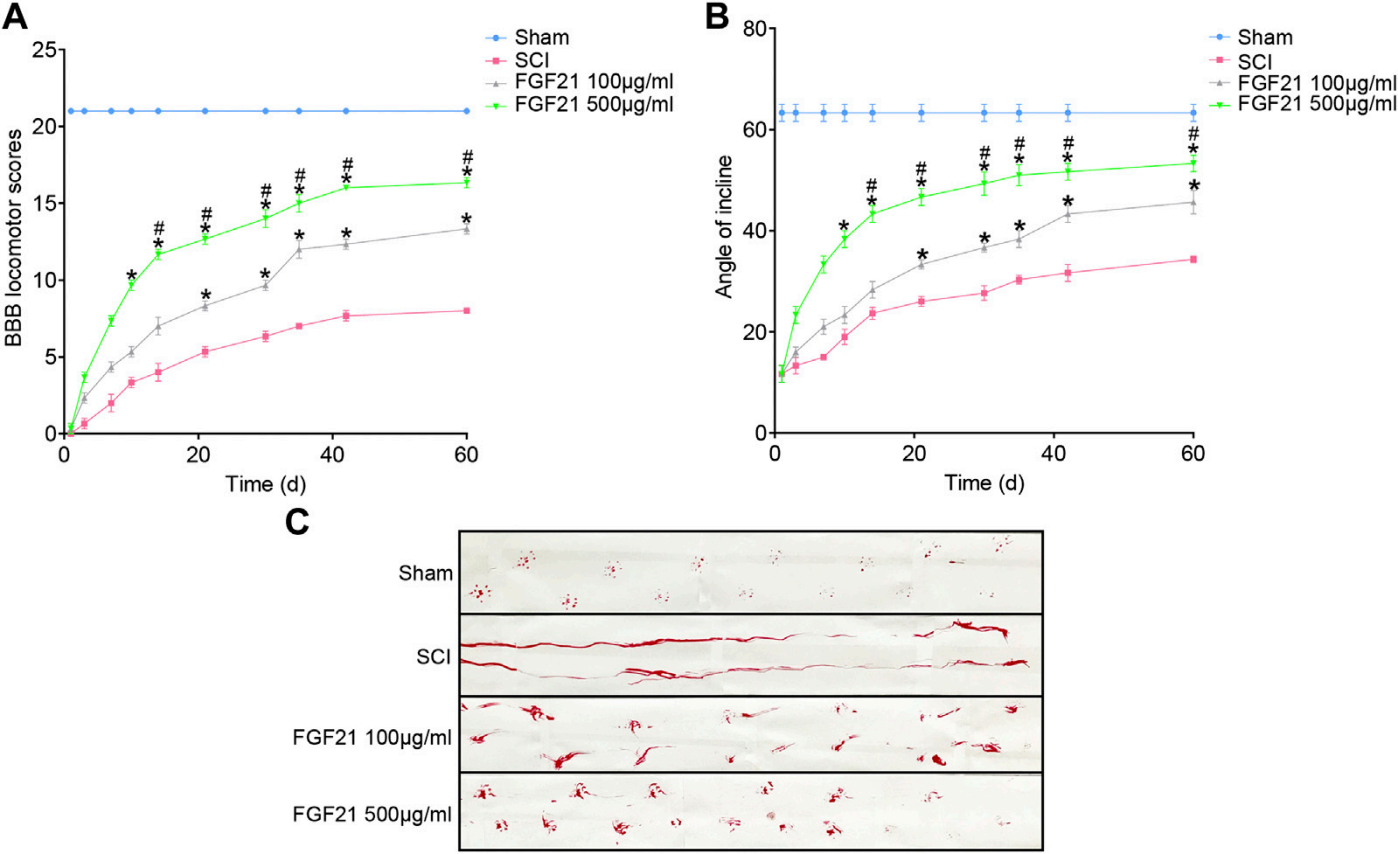
FGF21 treatment facilitates locomotor function after SCI **(A,B)** Assessment of BBB scale and inclined plane test for each group. BBB score of 21 represented by the sham group is regarded as full score. Representation of data is mean values ±SEM, *n* = 6, “*” and “**” represent *p* < 0.05 or *p* < 0.01 vs the SCI group, “#” represent *p* < 0.05 vs the 100 μg/ml FGF21 group **(C)** Image of footprint analyses of sham, SCI group, 100 μg/ml FGF21 group and 500 μg/ml FGF21 group.

Further, using BBB scale and inclined plane test, we found that FGF21 treated groups exhibited improved locomotor function, when compared with SCI group (F = 4.229 in BBB scale, F = 4.158 in inclined plane test. Figures 2A,B). Consistently, by footprint analysis, FGF21 treated groups showed improved functional recovery of SCI when compared with SCI group (Figure 2C). Collectively, these results demonstrate a therapeutic effect of FGF21 systemic administration on SCI repair.

### FGF21 Treatment Promotes the Survival of Neurons and the Improvement of Tissue Density

**FIGURE 3:**
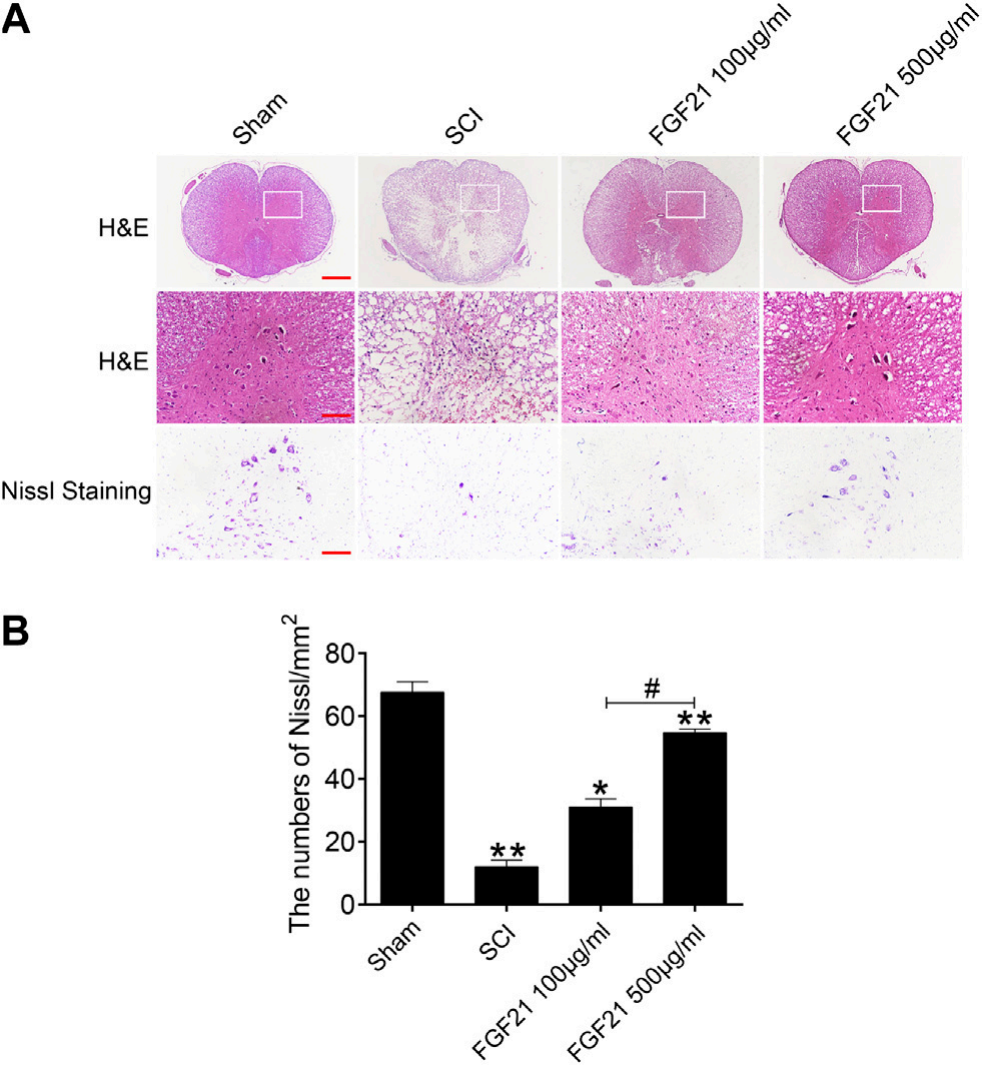
FGF21 treatment improves the tissue structure and number of Nissl bodies **(A)** Images captured after H&E staining and Nissl staining. The images showed the whole cross section of spinal cord, scale bar = 100 μm. The part restricted by a rectangle represents region with high power images, scale bar = 100 μm. The scale bar of Nissl staining images are 100 μm **(B)** Statistical graph of quantification of Nissl staining data from **(A)**. Representation of data is mean values ±SEM, *n* = 6, “*” and “**” represent *p* < 0.05 or *p* < 0.01 vs the SCI or Sham group, “#” represent *p* < 0.05 vs the 100 μg/ml FGF21 group.

H & E staining and Nissl staining were performed to examine the structure of the spinal cord tissues. The staining of sham group showed a complete organizational appearance with quite a few large and medium-sized neurons. SCI group showed that the some tissues at SCI sites were damaged, accompanied with a massive reduction in the number of neurons. In FGF21 treated groups, small and sporadic voids were detected with no apparent necrosis. Noticeably, FGF21 treated group with administration of 500 μg/ml FGF21 group displayed a more complete organizational structure with significantly more neurons and Nissl bodies than 100 μg/ml FGF21 group and SCI group, indicative of a dose dependent effect (Figure 3).

### FGF21 Treatment Increases the Expression of NeuN, GAP43 and NF200, and Deceases the Expression of GFAP

**FIGURE 4:**
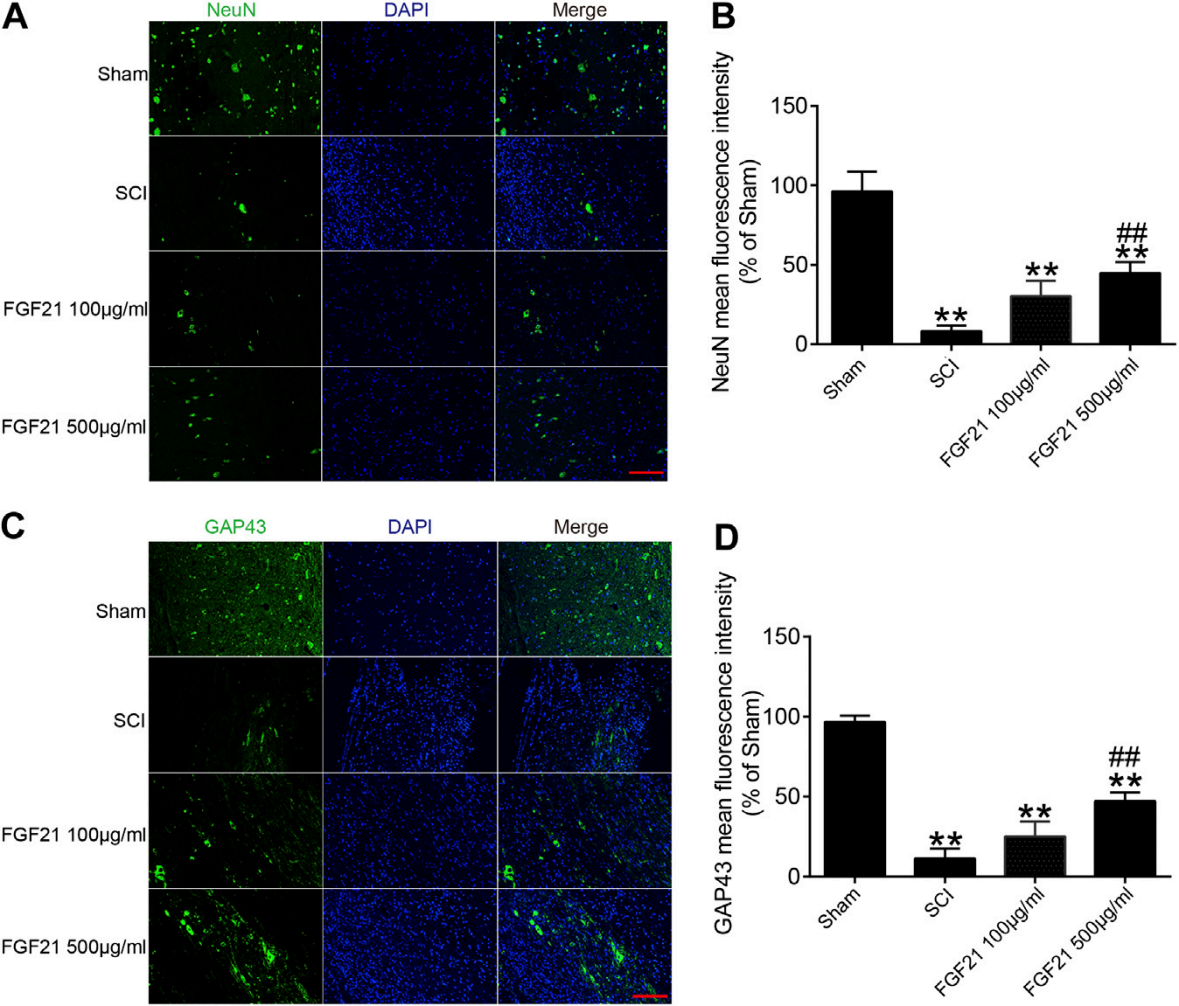
FGF21 treatment increases the expression of NeuN and GAP43 **(A,C)** Image of Immunofluorescence staining of NeuN and GAP43 in spinal cord lesions for each group. The bright green dots are positively stained neurons marked with obvious NeuN and GAP43; respectively. DAPI (blue) is applied to mark nuclei. scale bar = 100 μm **(B,D)** Analysis of mean fluorescence intensity. Representation of data is mean values ±SEM, *n* = 6, “**” represent *p* < 0.01 vs the SCI or Sham group, “##” represent *p* < 0.01 vs the 100 μg/ml FGF21 group.

Next, the protein expressions of NeuN and GAP43 were used to evaluate neural regeneration. The immunofluorescence staining of NeuN showed that more green fluorescence signals in FGF21 treated groups, when compared with the SCI group (Figures 4A,B). Similarly, the immunofluorescence staining of GAP43 showed that more green fluorescence signals in FGF21 treated groups, when compared with the SCI group (Figures 4C,D). These results indicated that FGF21 had a positive effect on the survival and regeneration of axon.

**FIGURE 5:**
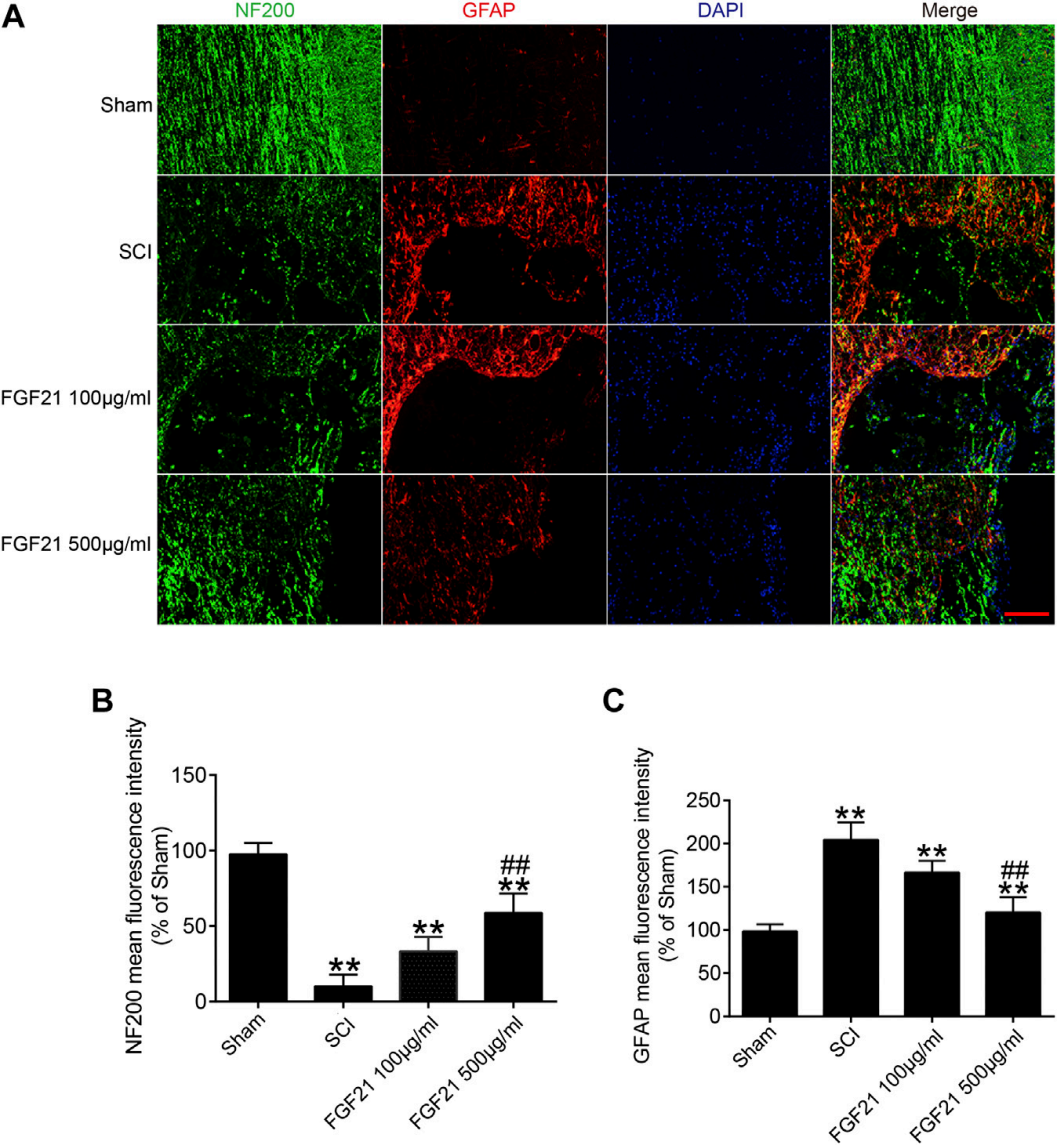
FGF21 treatment increases the expression of NF200, and deceases the expression of GFAP **(A)** NF200 and GFAP staining in the sham, SCI group, and FGF21 treated groups. The positive bright green dots represent NF200 staining, while the bright red dots represent GFAP staining. DAPI (blue) is in application to mark nuclei, Scale bar = 100 μm **(B,C)** Quantification of the NF200 and GFAP positive staining is reflected by mean fluorescence intensity. Representation of data is mean values ±SEM, *n* = 6, “**” represent *p* < 0.01 vs the SCI or Sham group, “##” represent *p* < 0.01 vs the 100 μg/ml FGF21 group.

Further, the immunofluorescence staining of NF200 showed that the expression of NF200 was higher in FGF21 treated groups, when compared with SCI group (Figure 5). These results suggest that the systemic administration of FGF21 has the potential to promote or maintain the regeneration of axon expanded over the scar boundary after SCI.

In addition, protein GFAP is used to determine the formation of the glial scar after SCI. Consistently, the immunofluorescence staining of GFAP revealed that the GFAP expression were lower in FGF21 treated groups when compared with SCI group. These data suggest that FGF21 systemic injection could inhibit glial scar formation of SCI (Figure 5). Taken together, these results indicate that FGF21 treatment inhibits formation of glial scar and promotes axon regeneration expanded over the scar boundary.

### FGF21 Administration Attenuates Autophagy Induced by SCI

**FIGURE 6:**
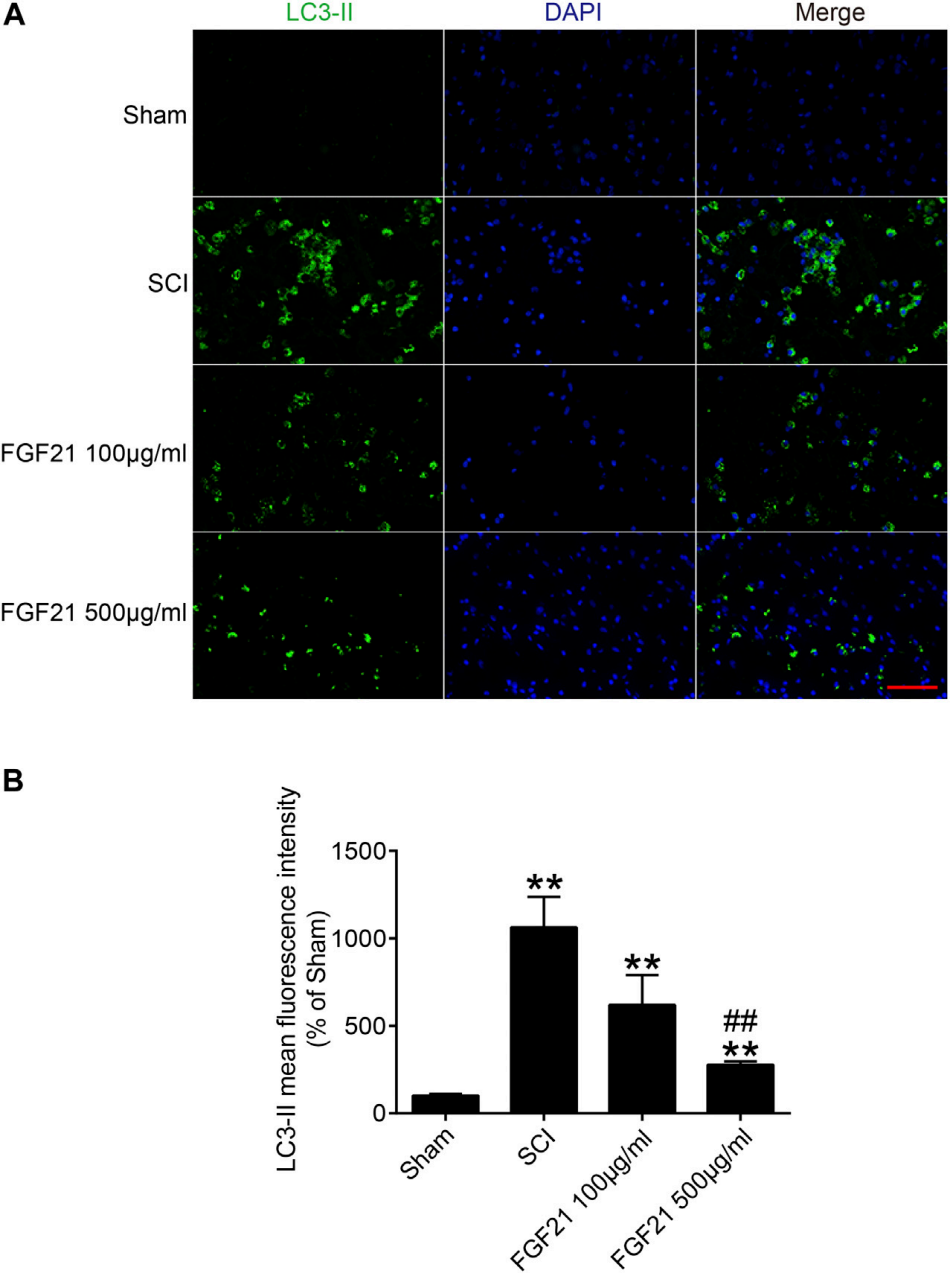
FGF21 administration results in attenuation of SCI-induced autophagy **(A)** The immunofluorescence staining for LC3-II in Sham, SCI group, and FGF21 treated groups. Green fluorescence represents LC3-II. DAPI (blue) is applied to stain nuclei. Scale bar = 50 μm **(B)** Quantitation of the Immunofluorescence staining results of LC3-II by mean fluorescence intensity. Representation of data is mean values ±SEM, *n* = 6, “**” represent *p* < 0.01 vs the SCI or Sham group, “##” represent *p* < 0.01 vs the 100 μg/ml FGF21 group.

To further explore whether the mechanism of FGF21’s *in vivo* effect may involve regulating autophagy. We applied immunofluorescence staining to detect the protein expression of LC3-II, an autophagy marker, and found that LC3-II protein expression was upregulated in SCI (Figure 6). Interestingly, the expression level LC3-II protein was decreased in FGF21 treated groups in a dose dependent manner, when compared with SCI group. Collectively, our results indicate that FGF21 is able to attenuate the SCI-induced autophagy, consistently with its *in vivo* therapeutic effect on the functional recovery of SCI (Figure 6).

## Discussion

In this study, using systemic administration of FGF21 to rat model of SCI, we have shown that FGF21 promotes the functional recovery of SCI and inhibits SCI-induced autophagy, suggesting a promising potential of FGF21 systemic administration in the therapy of SCI repair for the first time.

FGF21 is generally considered to be an effective metabolic regulator (Keipert et al., 2020; Zarei et al., 2020). There are few reports on the role of FGF21 in mediating neuroprotection and nerve regeneration. For instance, decreased FGF21 signal has been shown not only to reduce the number of new axons formed in the damaged loci, but also to alter the molecular structure of axons(Douris et al., 2017; Lovadi et al., 2017; Xiao et al., 2019). However, the role of FGF21 systemic administration in SCI repair remains hitherto unknown. In this study, we showed the effect and underlying molecular mechanism of FGF21 in mediating neuroprotection and nerve regeneration after SCI. Interestingly, we have found that FGF21 has a potent neuroprotective effect on SCI, manifested in the recovery of motor function, inhibition of neuronal death, and promotion of axonal elongation. In order to further explore the protective mechanism of FGF21 on neurons, we have investigated the effect of FGF21 on the autophagy.

After SCI, a microenvironment of ischemia, hypoxia and inflammatory infiltration is formed at the injury site (Fan et al., 2020; Kumar et al., 2020; Qian et al., 2020). Subsequently, lysosomal damage and dysfunction can occur within affected cells, leading to autophagy flux defects, accumulation of autophagosomes, initiation of long-term large-scale autophagy, and active cell death, which is not conducive to the survival of neurons (Bae et al., 2019; Rehorova et al., 2019). Inhibition of excessive autophagy activation by pharmacological intervention has been suggested to be a new tactic to reduce neuronal death (Lee et al., 2020). Compared with the short-term stress responsees such as oxidative stress and apoptosis, autophagy exists in the whole process of SCI (Cortes et al., 2014; He et al., 2016; Vahsen et al., 2020). Consistently, we found that autophagy-related protein level LC3 II was significantly induced in SCI, and whereas FGF21 attenuated SCI-induced autophagy and improves the functional recovery of SCI.

Previous studies have shown that SCI can lead to the death of a large number of neurons, the rupture of axons, and the formation of dense glial scar to block axon growth and elongation (Wang et al., 2019; Ma et al., 2020; Tran et al., 2020). These pathological changes could result in severe motor sensory dysfunction (Su et al., 2019). There is increasing evidence that autophagy is critically involved in the death of nerve cells in the central nervous system injury conditions (Pivtoraiko et al., 2009; Chen et al., 2017; Yan et al., 2019). Since it is difficult for neurons to regenerate, the best solution for neuron injury is to protect neurons and reduce excessive autophagy in neurons (Tsumuraya et al., 2015). Therefore, blocking autophagy cell death by FGF21 may represent an effective strategy to inhibit neuron death and promote axonal elongation after SCI.

**FIGURE 7:**
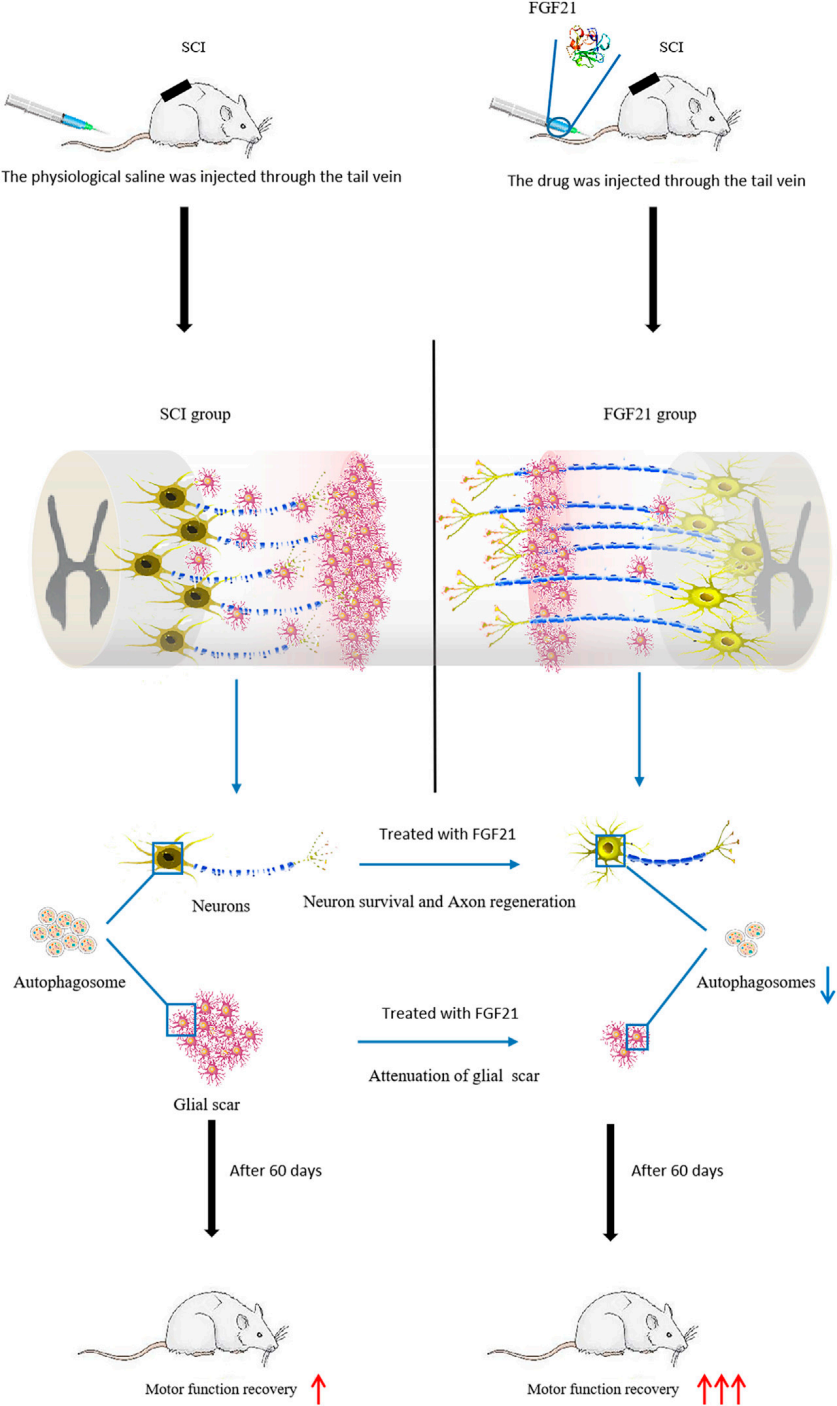
Injection of FGF21 inhibits autophagy and promotes motor function recovery.

Taken together, our data indicate that systemic administration of FGF21 can effectively improve the functional recovery after SCI, reduce neuron death, and inhibit autophagy, and thus imply that FGF21 may serve as a potential therapeutic molecule for SCI repair (Figure 7).

## Data Availability Statement

The raw data supporting the conclusions of this article will be made available by the authors, without undue reservation.

## Ethics Statement

The animal study was reviewed and approved by Animal Care and Use Committee of Wenzhou Medical College (wydw2014-0074).

## Author Contributions

SZ coordinated and carried out most of the experiments and data analysis, and participated in drafting the manuscript. YY and LY provided technical assistance. WY, JY, QW, and MC provided assistance on data analysis and manuscript preparation. SZ, HZ, XL, HD, HX, JX, and ZW supervised the project and experimental designs and data analysis. JX and ZW revised the manuscript. All authors approved the final manuscript.

## Funding

This study was partly funded by a grant the National Natural Science Funding of China (81802235), Zhejiang Medical and Health Science and Technology Plan Project (2021KY212), and Wenzhou Basic Science Research Plan Project (Y2020050, Y2020388, Y20190265).

## Conflict of Interest

The authors declare that the research was conducted in the absence of any commercial or financial relationships that could be construed as a potential conflict of interest.
